# The subcellular organisation of *Saccharomyces cerevisiae*

**DOI:** 10.1016/j.cbpa.2018.10.026

**Published:** 2019-02

**Authors:** Daniel JH Nightingale, Aikaterini Geladaki, Lisa M Breckels, Stephen G Oliver, Kathryn S Lilley

**Affiliations:** 1 Cambridge Centre for Proteomics, Department of Biochemistry, University of Cambridge, Tennis Court Road, Cambridge, CB2 1QR, United Kingdom; 2 Cambridge Systems Biology Centre, Department of Biochemistry, University of Cambridge, Tennis Court Road, Cambridge, CB2 1GA, United Kingdom; 3 Department of Genetics, University of Cambridge, Downing Street, Cambridge, CB2 3EH, United Kingdom

## Abstract

Subcellular protein localisation is essential for the mechanisms that govern cellular homeostasis. The ability to understand processes leading to this phenomenon will therefore enhance our understanding of cellular function. Here we review recent developments in this field with regard to mass spectrometry, fluorescence microscopy and computational prediction methods. We highlight relative strengths and limitations of current methodologies focussing particularly on studies in the yeast *Saccharomyces cerevisiae*. We further present the first cell-wide spatial proteome map of *S. cerevisiae*, generated using hyperLOPIT, a mass spectrometry-based protein correlation profiling technique. We compare protein subcellular localisation assignments from this map, with two published fluorescence microscopy studies and show that confidence in localisation assignment is attained using multiple orthogonal methods that provide complementary data.

## Highlights

• Protein subcellular localisation is essential for cellular homeostasis.
• Factors governing protein localisation are poorly understood.
• Various different methods exist to study this process.
• Recent studies have captured ever higher resolution localisation information.
• Orthogonal methods should be used to gain a holistic view of protein localisation.

**Current Opinion in Chemical Biology** 2019, **48**:86–95

This review comes from a themed issue on **Omics**

Edited by **Ileana M Cristea** and **Kathryn S Lilley**

For a complete overview see the Issue and the Editorial

Available online 29th November 2018

**https://doi.org/10.1016/j.cbpa.2018.10.026**

1367-5931/© 2018 The Authors. Published by Elsevier Ltd. This is an open access article under the CC BY license (http://creativecommons.org/licenses/by/4.0/).

## Introduction

The subcellular location of a protein is of paramount importance, dictating the environment in which it can function. It is vital to the plethora of subcellular mechanisms that underpin the correct functioning of cells that proteins are precisely located where they can interact with appropriate binding partners including other proteins, nucleic acids and metabolic substrates. The subcellular location of proteins in many cases is highly dynamic, with some proteins that traffic continuously and others that selectively localise to specific subcellular compartments. Moreover, many proteins re-localise in response to external and internal signals. Aberrant subcellular localisation of proteins has been implicated in various diseases including cancer, obesity and several protein mis-folding diseases [1, 2, 3, 4, 5, 6].

The factors that control where a protein is located are manifold and remain poorly understood. In some cases signals encoded in the primary sequence of the protein control its final destination. This may be based on physicochemical characteristics, for example the mitochondrial targeting sequences that direct nuclear-encoded proteins to this organelle [7]; or sequence tags such as C-terminal HDEL, KDEL, or variant motifs that signal retention in the ER [8,9]. In other cases proteins are trafficked to a subcellular niche based on interactions with protein partners, as is the case for the protein kinase PRAK [10]. This protein contains a nuclear localisation sequence, but its localisation is determined by which isoform of its upstream kinase p38 it is bound to as only one isoform interacts with nuclear import machinery, affecting its localisation. A protein’s destination is also influenced by post-transcriptional modifications such as alternative splicing. Different isoforms of the leucine aminopeptidase Lap3 exhibit different subcellular localisations, with the canonical isoform being located at the mitochondrion and a truncated isoform localised elsewhere [11^••^]. Localisation is significantly influenced by post-translational modifications such as phosphorylation, which affects the localisation of transcription factors to the nucleus in multiple biological systems [12, 13, 14], and addition of glycophosphatidylinositol anchors that anchors proteins to cellular membrane [15]. Finally, a protein’s final location may be dictated by the site of its translation as some transcripts are localised to an organelle before translation, sometimes by specific protein families such as the RNA-binding PUF protein family, members of which can transport transcripts to the ER [16] and mitochondria [17].

The baker’s yeast *Saccharomyces cerevisiae* is an attractive model eukaryotic system in which to study protein subcellular localisation. It has been employed to address a host of biological questions, due to its well-annotated genome, genetic tractability, ease and scalability of culture and the homology of some of its proteins to those of higher organisms [18]. The wealth of techniques and resources available have established *S. cerevisiae* as a model organism of choice. In the two decades since its genome sequence was published [19] a host of resources have become available, including the organism-specific *Saccharomyces* Genome Database (SGD—www.yeastgenome.org) [20]. The SGD also contains repositories of Gene Ontology (GO) [21,22] cellular compartment (CC) information regarding protein subcellular information. There are also numerous yeast strains available, including organism-specific tagged libraries [23, 24, 25,26^•^,27^•^], of which a set of systematically GFP-tagged ORF libraries for protein subcellular localisation studies are particularly useful [25,26^•^,27^•^].

**Figure 1:**
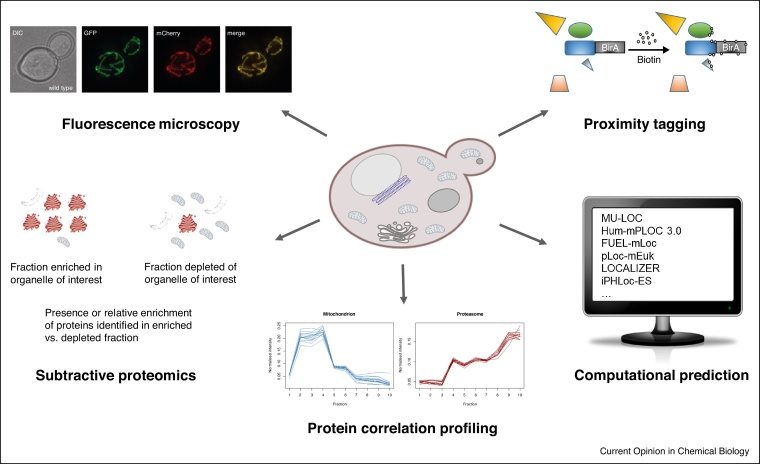
A summary of the main approaches that can be used to study subcellular protein localisation in *S. cerevisiae* and other organisms, as reviewed in this article. The fluorescence microscopy figure is reproduced from Ref. [81].

In two recent publications it was suggested that up to half of the proteome of a eukaryotic cell resides in multiple subcellular locations [11^••^,28^••^]. In many cases the reasons for a protein’s multiple localisation and its mechanistic purpose are unknown. Although this phenomenon has been the focus of much study, it is clear that our knowledge of factors that dictate a protein’s destination in a cell is far from complete. There are multiple approaches to the study of subcellular protein localisation that aim to address this issue (illustrated in Figure 1). In this review, we firstly discuss recent developments in methods to study subcellular protein localisation, focussing on *S. cerevisiae* and in respect of the themes listed below.

1 Computational predictions
2 Mass spectrometry approaches
  i Proximity tagging
  ii Subtractive proteomics
  iii Protein correlation profiling approaches
3 Fluorescence microscopy

Secondly, we compare and contrast results from a new study presented here that creates a cell-wide map of yeast proteins using quantitative proteomics coupled with cell fractionation, with data gleaned from orthogonal methods using techniques described above. We show that the combination of multiple methods gives confidence to our knowledge of the subcellular locations of proteins, but also highlights limitations of modern methodologies. Finally, we provide evidence from new and old studies that the majority of yeast proteins in a cell reside in multiple places. This adds to speculation of the high dynamicity of the spatial proteome and potentially supports previous observations of proteins fulfilling multiple different functions based on where they are located within the same cell type (so-called ‘moonlighting’ [29^•^]).

### Computational prediction methods

As already stated, the ultimate destination of a protein in a cell is often locked into its primary sequence. Several machine learning classifier-based prediction tools for spatial proteomics analysis exist to predict protein residency based on inherent, experimenter-provided or publicly available data, within fluorescence microscopy-based datasets [30,31] and correlation profile-based datasets [32, 33, 34, 35, 36]. A host of purely computational tools are also available for the prediction of protein subcellular localisation, which are capable of predicting organelle residency for a protein-based solely on sequence or other features (reviewed in Ref. [37]). For instance one tool, SignalP [38], uses artificial neural networks to predict the presence of signal peptides that direct proteins through the secretory pathway, whilst distinguishing them from N-terminal transmembrane helices. Proteins do not always contain signal sequences in their primary sequence that make them obvious candidates for localisation to a given organelle, so often these programs are based on machine learning algorithms that train classifiers to predict protein localisation to organelles based on domain information, suspected transit peptides, amino acid frequencies, GO CC information or other sequence information. These are available for a multitude of biological systems and can be used to predict a single [39,40] or multiple protein locations [41,42], discriminating between distinct suborganellar localisations [43,44] as well as predicting the localisation of proteins secreted by pathogens [45,46]. These tools are typically reported to perform well with test data. In the case of reference [44] whose focus is on submitochondrial localisation, the predictive performance for the reported tool reports sensitivity of >84%, and specificity and accuracy both of >94%, for prediction of proteins to be at the mitochondrion. For submitochondrial locative prediction these parameters were lower, but were all >71%. Further, for Ref. [45], locative prediction to chloroplast, mitochondrion and nucleus using LOCALIZER reports specificity of over 79%, sensitivity of 60% and over, and accuracy of 73% and over.

### Mass spectrometry methods

Over the past two decades a variety of proteomics approaches have emerged that couple isolation, enrichment or labelling of subcellular niches with quantitative mass spectrometry to determine protein location. Recent developments in these approaches are discussed subsequently.

#### Proximity tagging

Several recent studies have reported the use of proximity tagging methods to study subcellular protein localisation in *S. cerevisiae*. The premise of these methods is that an enzyme capable of protein biotinylation, typically a biotin ligase (BirA) or ascorbate peroxidase (APEX), is tagged in-frame in a specific ORF of interest and expressed *in vivo.* Given addition of exogenous biotin (for BirA [47]), or hydrogen peroxide and biotin-phenol (for APEX2 [48]), a biotinylation reaction occurs that results in labelling of proximal and interacting proteins at lysine and tyrosine residues respectively, that were within a specific radius of the protein of interest *in vivo*. The proximal proteins are affinity-purified by virtue of their biotinylation and characterised by methods including mass spectrometry. Several homologous BirA enzymes for proximity labelling have been published, including one from *Escherichia coli* (used in the BioID technique [49]), one from *Aquifex aeolicus* (used in BioID2 [50]) and a recently published variant from *Bacillus subtilis* (used in BASU [51]). Two other enzymes, TurboID and miniTurbo, have recently been developed based on directed evolution of *E. coli* BirA in *S. cerevisiae*, that have faster labelling kinetics and are smaller than the original *E. coli* enzyme [52]. All of these ligases differ in size and biotinylation speed, with TurboID being the fastest BirA published to date [52]. The biotinylation radii of these methods vary, from ∼20 nm in the case of APEX [48], to >10 nm for the BioID2 technique, given inclusion of a linker peptide to increase the radius [50]. Some of these approaches have been used to infer suborganelle proteomes, including for non-membrane delineated regions, in systems other than *S. cerevisiae* [53,54,55^•^].

A recent study [56^•^] used the BioID method in *S. cerevisiae* in combination with triplex SILAC labelling [57] and LC–MS/MS analysis, to infer changes in the proximate proteins of an important scaffold protein constituent of the ribosome (Asc1p) under multiple stresses. SILAC labelling enabled true proximate proteins to be inferred by relative enrichment of proteins in the scaffold protein biotinylation channel relative to two negative control pulldown channels. APEX has been used successfully in multiple biological systems, including recently the fission yeast *Schizosaccharomyces pombe* [58]. The approach has further been demonstrated in a non-directed proof-of-concept experiment in *S. cerevisiae* that was contingent on the absence of an intact cell wall. Exogenous hydrogen peroxide and biotin-phenol were demonstrated to traverse the cell membrane and biotinylation occurred in a strain expressing APEX2 alone, being expressed from an episomal plasmid and under the control of a strong promoter [58].

#### Subtractive proteomics

Protein localisation may be studied by characterising the simple presence or absence, or the relative difference in abundance of proteins from preparations of one, or several, organelles. This is based on the premise that proteins more enriched in an organelle fraction of interest than a contaminant organelle fraction are more likely to be localised to the organelle of interest. Enrichment is often based on unique physical properties of the organelles in question. This approach can suffer from lack of purity of the organelle preparation, which other organelles with similar physical properties may contaminate, thus complicating the analysis. In addition, proteins that can be present in more than one subcellular location are not distinguished by this approach which aims to study an organelle in isolation and does not faithfully recapitulate what occurs within the intact cell.

Nevertheless, subtractive methods have been performed extensively in *S. cerevisiae* to characterise the residents of multiple organelles. For example, the vacuolar proteome was defined using an approach where true residency was inferred by quantitative enrichment of proteins, using iTRAQ [59], and comparing enriched versus crude vacuolar membrane preparations [60]. The plasma membrane proteome has been defined using 2D-PAGE and mass spectrometry, both in the presence and absence of cell wall stress [61]. A recent study investigated protein constituents of the tubular ER in *S. cerevisiae* using immuno-isolation of an epitope-tagged version of a tubular ER protein coupled with a quantitative mass spectrometric comparison, using triplex dimethyl labelling [62,63]. Several studies have focussed on the mitochondrion, some of which used orthogonal qualitative enrichment methods and defined the overlap of identified proteins as the true mitochondrial proteome [64,65]; suborganellar compartments of the mitochondrion in isolation, including the outer membrane [66] and also the intermembrane space (IMS) using quantitative proteomics [67]. Two recent studies have also utilised more sophisticated approaches involving multiple quantitative mass spectrometry methods and suborganellar preparations to map the complement of the submitochondrial proteome [68^••^,69^•^], although in some cases, with limited control of incorrect assignment of contaminating proteins from other organelles.

#### Protein correlation profiling methods

Several studies have utilised quantitative, protein correlation profile-based approaches to map the spatial proteome on a more cell-wide scale. These methods are predicated on the observation that when cell lysates are fractionated, proteins that are localised to the same subcellular location will behave in a similar way [70]. Organelle proteins sediment in a manner characteristic of the organelle in question, which importantly is different from proteins localised to other organelles that are sampled within the same experiment. Co-fractionation was originally characterised using enzyme activity assays [70] but now uses quantitative mass spectrometry.

Several studies focusing on mammalian systems have made use of protein correlation profiling approaches. Some have carried out subcellular fractionation using differential centrifugation approaches [34,35]. Using a variety of quantitation approaches these studies have led to partial cell maps being produced with, in some cases, limited subcellular resolution. Equilibrium centrifugation has also been used to fractionate cellular compartments [36], again with partial cell coverage.

A more rigorous and holistic approach has been afforded by hyperplexed Localisation of Organelle Proteins by Isotope Tagging (hyperLOPIT) [11^••^,28^••^,^71^], a methodology that combines biochemical fractionation of cell lysates by isopycnic density gradient centrifugation, high throughput mass spectrometric quantitation and machine learning [72]. After subcellular fractionation, proteins are tryptically digested and differentially labelled with TMT tags [73] before pre-fractionation and analysis by LC–MS/MS. The high multiplexing capability of TMT, coupled with the exquisite resolution offered by density gradient separation, enables generation of highly resolved spatial maps. This technique has been used to map the spatial proteome of the E14TG2a mouse embryonic stem cell line [11^••^] and the human U-2 OS cell line [28^••^], both with unprecedented resolution. This method has the highest subcellular resolution of any MS-based method to date [Gatto *et al*., this issue]. Importantly this method is able to determine proteins residing in multiple compartments and large protein complexes.

### Fluorescence microscopy

Fluorescent protein tagging has emerged as a powerful tool to visualise the localisations of individual proteins by microscopy on a cell-wide scale in *S. cerevisiae* [25]. Variants of this approach have been extensively used to map the spatial proteome under various conditions of stress [30,31,74,75]; each time producing a variant reference map of subcellular protein localisation under non-perturbed conditions. These studies employed the same GFP library originally published in Ref. [25] in which 6,029 ORFs were C-terminally tagged; or variations of this library in some cases containing housekeeping proteins tagged with different fluorescence proteins to carry out relative expression studies. Overall 4156 proteins gave GFP signal above background in the original study [24]. Of 5330 strains queried in another study [74], over 1800 yielded no localisation information as protein expression levels were insufficient. Furthermore, strains expressing 187 tagged proteins were systematically removed from Ref. [74] due to their requirement of an uninterrupted C-terminus for correct localisation. Whilst powerful, a limitation of such methods is that the generation of libraries is time-consuming and labour-intensive. Furthermore, it is not always possible to assign protein localisation to a discrete subcellular location, due to localisation uncertainty, illustrated by the use of the descriptors ‘ambiguous’ and ‘punctate’ in some of the aforementioned studies. Limitations to the resolution of the microscopy platforms used also mean that it is often not possible to assign protein localisation to particular protein complexes or suborganellar locations.

A recent study has described the use of a new strategy (SWAp-Tag) in *S. cerevisiae* which facilitates the manipulation and generation of systematic organism libraries in a much more routine manner [26^•^]. This method was employed to generate multiple new fluorescent protein tag libraries for microscopy-based mapping studies; one of which contains a tag that is C-terminal, and several others of which contain a tag that is N-terminal, to the ORF [26^•^,27^•^]. Included within the N-terminal libraries are two in which the protein is predicted to contain N-terminal targeting sequences (to the secretory pathway and mitochondria). The tag has been engineered to contain targeting sequences to these organelles, enabling visualisation of protein subcellular localisations that would not have been possible due to the interruption of the targeting sequence by the tag in previous fluorescent protein libraries [25]. It is worth noting that the targeting sequences within the tags are endogenous, but not specific to the proteins under investigation. The new localisations should therefore be viewed as solely predictive as they are not expressed with their own native targeting sequences.

### Comparison of hyperLOPIT data with orthogonal *S. cerevisiae* subcellular data

Interrogation of published data for yeast protein subcellular localisation datasets highlights two issues. Firstly, many studies [7,60,61,63, 64, 65, 66, 67,68^••^,69^•^] only provide subcellular localisation data regarding a single subcellular niche, meaning that if a protein is located in more than one place, only one location is reported. Consequently, important information regarding a protein’s ability to traffic between and potentially function in a variety of subcellular niches is lost. Secondly, interrogation of published datasets that have been created using orthogonal methods reveals poor overlap in some cases. This is true for the data presented in Ref. [69^•^], where some assignments to a mitochondrial subcompartment are non-concordant with previous fluorescence studies [25,74] including proteins that are assigned to the cell periphery by the microscopy studies; and some proteins that are predicted to reside at the plasma membrane in Ref. [61] but are predicted to reside at multiple other locations in the fluorescence studies [25,74]. A comparison was also made by Dénervaud *et al.* [31] of results from their study compared with a study by Tkach *et al*. [75] interrogating a stress condition that was in common between their two studies, that used orthogonal microscopy-based methods to study protein subcellular localisation. Dénervaud *et al*. captured time-lapse films of protein localisation during culture and carried out localisation analysis in an automated fashion, whereas Tkach *et al*. captured localisation at a single time point and carried out localisation analysis manually. Using their method, Dénervaud *et al*. found 81 more re-localisation events in response to the same stress than were observed in Ref. [75] (31 re-localisations). In addition Chong *et al.* [30] performed several comparisons of the results of their fluorescence study, in which protein localisation was predicted using machine learning, with those of Huh *et al.* [25], in which protein localisation was assigned manually, and found for example a 9% non-concordance in proteins that were predicted to reside in a single location. Chong *et al.* further compared their data with the work of Tkach *et al*. who used one of the same stresses, to benchmark their protein re-localisation analysis method, finding that approximately half of their protein re-localisation predictions were in agreement with the Tkach study.

**Figure 2:**
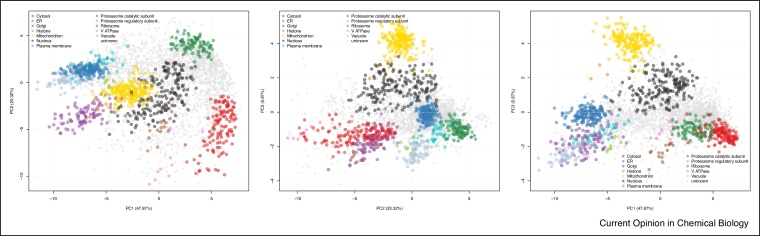
Two-dimensional principal components analysis (PCA) plots reveal the extent of assignment of proteins of unknown localisation to subcellular organelles and niches using the hyperLOPIT technique in *S. cerevisiae*. In the PCA plots, each point represents a single protein group that was observed and fully quantified in our experiment. Good resolution of twelve separate subcellular organelles and niches is observed, encompassing the major subcellular organelles of *S. cerevisiae*. This includes suborganellar resolution such as the V ATPase and the two proteasome subunits. Plotting principal components 1 and 2 (left panel) reveals resolution of most organelles, whereas plotting principal components 2 and 3 highlights resolution of the denser organelles, including the nucleus (black) and mitochondrion (yellow). Plotting components 1 and 3 (right panel) reveals resolution of the secretory pathway organelles (lower right hand quadrant) that are not as easily resolved in the other 2 panels.

No comprehensive comparison of data acquired using a truly orthogonal method of capturing cell wide protein localisation with data arising from high throughput microscopy exists to date. Unlike other organisms, there is no data resulting from correlation profiling methods for *S. cerevisiae*. We therefore set about applying the hyperLOPIT methodology to investigate the spatial proteome of this organism (Supplementary methods). We performed the experiment as described in Ref. [76] using the culture conditions from Huh *et al*. [25] that were also common with the study of Breker *et al*. [74]. We carried out four biological replicate hyperLOPIT experiments, two of which contained nuclear preparations and two did not, as these variant experiments provided complementary organelle resolution. We concatenated the datasets using a method described in Ref. [77] to obtain 2846 common protein groups (Supplementary data 1) and classified organelle residency of proteins by SVM as described previously [11^••^,28^••^,^71^] (see Supplementary data 2 for SVM training data). We resolved 12 organelles, subcellular compartments and large protein complexes (collectively referred to as ‘niches’) within our spatial proteome map. Of importance and in common with two previous hyperLOPIT studies [11^••^,28^••^], after assignment of proteins that localise to a subcellular niche we observed that less than half of the proteome was predicted to localise to a single subcellular location, underlining the dynamic nature of the spatial proteome in multiple biological systems (Figure 2 and Supplementary data 3).

As the culture conditions were shared with two previous studies [25,74], a comparison of differences in protein localisation assignment between our results and these studies could be made. (Supplementary data 4 and 5). Organelle descriptors present in our study, including Golgi apparatus, ribosome, plasma membrane, cytosol and proteasome were missing from one or both of the microscopy-based studies. For the most part these represent organelles that may be more easily separated based on density, and more difficult to distinguish using the microscopy platforms employed. Conversely descriptors included in the fluorescence microscopy studies, such as cell periphery, bud, spindle pole and broad subcompartments such as the late Golgi were missing from our data. They most likely do not differ sufficiently in density to be resolved in this experiment but are more easily observed by microscopy.

Despite the fact that we could only compare proteins that were common between our data and these studies, we observed high agreement in assignment between our study and the fluorescence microscopy studies for proteins belonging to some subcellular locations. For example, the mitochondrion showed 95.5% agreement with [25], and 89.4% agreement with [74]. For other proteins that may be dynamically distributed between multiple different organelles such as those which belong to the secretory pathway, the difference in the level of agreement was more varied. This was true for the vacuole (57.1% agreement with [25], 48.6% agreement with [74]), ER (74.1% agreement with [25], 74.2% agreement with [74]) and plasma membrane (compared with terms including ‘cell periphery’—60.9% agreement with [25], 69.2% agreement with [74]). For non-concordant assignments many were assigned to other parts of the secretory pathway. For instance, 23.2% of proteins that do not agree in vacuole assignment are assigned by Ref. [25] to other parts of the secretory pathway such as the ER. Alternatively, this lack of concordance may be due to the proximity of some organelles within the cell to each other that may contribute to mis-assignment upon manual inspection of microscopy data. Indeed, the cortical ER and parts of cell periphery, or perinuclear ER and parts of the nucleus, may look similar by microscopy, thus proteins may be assigned to one or other of these organelles by imaging methods in a manner that is different from the one employed in hyperLOPIT. Some proteins that do not agree in assignment to the ER between hyperLOPIT and the two microscopy studies (4.6% for [25] and 4.3% for [74]) are assigned to the cell periphery or nuclear periphery by these imaging approaches. Comparing the plasma membrane and cell periphery, 21.7% of the proteins that do not agree between hyperLOPIT and [25] are assigned by hyperLOPIT to the ER. This effect is smaller but still valid for the comparison between hyperLOPIT and [74] (7.7% of proteins are predicted to be at the ER).

Comparing hyperLOPIT nuclear assignments with all subnuclear assignments in Ref. [25] and [74] revealed high percentages of concordance (86.3% and 74.8%, respectively). Of the proteins that are in disagreement, 17.6% of the hyperLOPIT nuclear predictions are assigned as cytosolic in Ref. [74] and 8.9% are assigned as cytoplasmic in Ref. [25]. Situations may also arise where one protein is annotated as localising to both locations in the fluorescence microscopy studies but only the nucleus in our hyperLOPIT data, although this is a negligible number (<1% in Ref. [25] and <4% in Ref. [74]). Comparing the cytosol in our study with cytoplasm [25] and cytosol [74], agreements vary from 53.7% to 88.3%, respectively, with a proportion predicted to be nuclear (7% in Ref. [74], 1.8% [25]) or localised to some part of the nucleus as well as the cytoplasm (7% in Ref. [74], 35.5% in Ref. [25]). Taken together, when comparing the hyperLOPIT nuclear and cytosolic locations to those reported by the other two studies for the same sets of proteins; each location alone, or both locations together, account for >90% of protein localisation assignments. These may be proteins that can be present at either of these two locations but which, for the most part, were localised to one localisation in each study. Comparing cytosolic assignment between hyperLOPIT and microscopy, especially for Ref. [25], the observed discrepancies may reflect differences in the ease of assigning proteins as being part of the cytosol when utilising these two orthogonal methods. Density-based separation approaches such as hyperLOPIT may lead to more easy assignment of protein to the cytosol as a subcellular location than is possible using microscopy.

Overall, our results map a relatively smaller proportion of the spatial proteome than the studies to which we compared our data. We argue that this may be due to the fact that the aim of those previous studies was to ascribe protein localisations as exhaustively as possible. Conversely, the aim of our study was to define the core proteins that localise to a single subcellular niche in nitrogen replete conditions, whilst preserving the dynamic character of the spatial proteome being sympathetic of proteins that reside in multiple locations. As such, our experiments provide data which are complementary to studies that have already been published.

## Conclusions

Subcellular protein localisation is vitally important, having widespread effects on the cell during organelle biogenesis and general cellular homeostasis. Indeed aberrant protein localisation has been implicated in numerous serious human diseases. The ability to understand the mechanisms governing this process at a deeper level will enhance our understanding of how cells function. There are several confounding factors, however, which make attaining high quality datasets in sufficient quantity to study protein localisation far from straightforward.

The past few years have seen exciting developments in multiple methods for the study of protein subcellular localisation in *S. cerevisiae*. Whilst some methods such as subtractive proteomics and fluorescence microscopy are relatively mature in their application to subcellular protein localisation in this system, the true potential and utility of others such as proximity labelling and whole-cell protein correlation profiling methods have yet to be demonstrated through acquisition of more and varied datasets. This is particularly true for the APEX2 approach in *S. cerevisiae* for which there are currently no large-scale experimental datasets. The new and promising TurboID, miniTurbo and BASU approaches should also be exploited to generate more and varied yeast datasets. New advances are being developed to address shortcomings in current methodologies and enable a more complete understanding of protein subcellular localisation than has been possible previously.

To gain a more complete picture of the *cis*-acting and *trans*-acting features of proteins that influence their location, it is necessary to collect as much data from as many cell types as possible using a variety of different methods that give precise and accurate information regarding this phenomenon. This has been exemplified by the comparison of the hyperLOPIT and two fluorescence microscopy studies which utilised the same culture conditions and yeast strain but in some cases obtained different and potentially valid variant subcellular locations for the same sets of proteins. The use of these methodologies for dynamic re-localisation experiments has already been demonstrated in a number of studies. We envisage, however, that collection of such datasets will facilitate the use of such methodologies to monitor dynamic protein subcellular re-localisation in response to stress, over developmental timescales and given perturbation in a more routine, fine-grained and higher resolution manner. We note that protein assignment to a subcellular location has often been performed manually and can be open to subjectivity, which may partially explain differences in localisation assignment between studies that use the same strains and experimental conditions. We thus argue that the focus should subsequently move on to analysis of spatial dynamics of the proteome in a more automated and unbiased way.

## Conflict of interest statement

Nothing declared.

## Data availability

All protein-level datasets are available in the R [78] Bioconductor [79] pRolocdata [72] package (https://bioconductor.org/packages/pRolocdata version 1.19.4) and can be interactively explored using the pRolocGUI [80] package (https://bioconductor.org/packages/pRolocGUI) or using the standalone online interactive app (https://proteome.shinyapps.io/yeast2018).

## References and recommended reading

Papers of particular interest, published within the period of review, have been highlighted as:

• of special interest
•• of outstanding interest

## Appendix A Supplementary data

Figure S1 Distribution of organelle marker proteins in a typical hyperLOPIT experiment, carried out in the absence of a nuclear preparation. Fractions are numbered from the least dense (1) to the most dense (22) fraction of the gradient. ‘C’ represents the separate cytosolic fraction. Organelle marker proteins, as well as the organelles which they represent, are labelled to the right of the Figure.
