# Supplementary material for: The subcellular organisation of Saccharomyces cerevisiae
Source: Curr Opin Chem Biol. 2019 Feb;48:86–95. doi: 10.1016/j.cbpa.2018.10.026 (PMC6391909; doi:10.1016/j.cbpa.2018.10.026)
Supplement: Supplementary file 6 [file mmc6.docx]

Supplementary Data 1. Quantitation information from the concatenated hyperLOPIT dataset, along with metadata describing the proteins observed in this dataset.

Supplementary Data 2. Marker proteins used for SVM classifier training in the *S. cerevisiae* hyperLOPIT experiment.

Supplementary Data 3. Determination of protein localisation assignment based on low throughput GO CC annotation from the Saccharomyces Genome database, with SVM score cut-offs indicated.

Supplementary Data 4. Comparison of the hyperLOPIT protein localisation assignment data with the data of Huh *et al.* (2003).

Supplementary Data 5. Comparison of the hyperLOPIT protein localisation assignment data with the data of Breker *et al.* (2013).
