# Supplementary material for: The subcellular organisation of Saccharomyces cerevisiae
Source: Curr Opin Chem Biol. 2019 Feb;48:86–95. doi: 10.1016/j.cbpa.2018.10.026 (PMC6391909; doi:10.1016/j.cbpa.2018.10.026)
Supplement: Figure S1 — Distribution of organelle marker proteins in a typical hyperLOPIT experiment, carried out in the absence of a nuclear preparation. Fractions are numbered from the least dense (1) to the most dense (22) fraction of the gradient. ‘C’ represents the separate cytosolic fraction. Organelle marker proteins, as well as the organelles which they represent, are labelled to the right of the Figure. [file mmc7.docx]

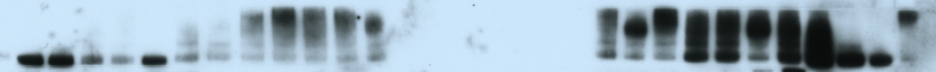

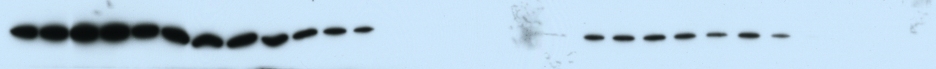

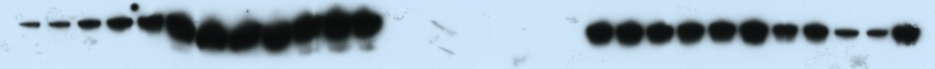

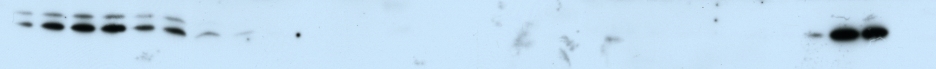

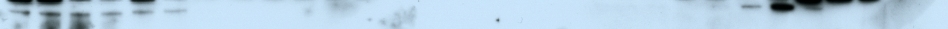

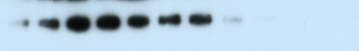

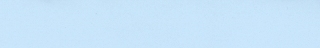

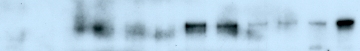

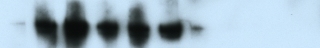


PEP1 (Golgi)

PMA1 (Plasma membrane)

PGK1 (Cytoplasm)

Histone H3 (Nucleus)

DPM1 (ER)

NSR1 (Nucleus)

COX2 (Mitochondrion)

9

3

1

2

4

5

6

7

8

10

11

12

13

14

15

16

17

18

19

20

21

22

C
