# Supplementary material for: The subcellular organisation of Saccharomyces cerevisiae
Source: Curr Opin Chem Biol. 2019 Feb;48:86–95. doi: 10.1016/j.cbpa.2018.10.026 (PMC6391909; doi:10.1016/j.cbpa.2018.10.026)
Supplement: Supplementary file 8 [file mmc8.docx]

Supplementary methods

Our hyperLOPIT experiments were performed according to reference [1]. The protocol used is outlined below.

*Cell culture and pre-treatment for lysis*

The yeast strain used in this study was BY4741 [2] harbouring GFP-tagged Hsp82p (*MAT***a** *HSP82-GFP::HIS3* *his3Δ1 leu2Δ0 met15Δ0 ura3Δ0*) [3] which was a kind gift from Professor Maya Schuldiner (Weizmann Institute of Science, Israel). The cells were cultured using SD-His media which was of the same composition as used in Breker *et al.* [4]. Cells (720 OD units per experiment) were cultured to OD_600_ of 0.6 at 30°C with constant shaking at 200 rpm. Our protocols for zymolyase digestion and lysis for isopycnic density gradient centrifugation were modified from a previous publication [5].

For zymolyase digestion, cells were resuspended in 25 mM Tris-HCl, pH 7.5, 10 mM Tris-(2-carboxyethyl) phosphine, at 5 OD units per mL, and incubated at room temperature for 5 minutes before being harvested. Cells were resuspended in spheroplasting medium (SD-His with the addition of 25 mM Tris-Cl, pH 7.5, 1.2 M sorbitol, 1 mM EDTA, pH 8.0 and cOmplete EDTA-free protease inhibitor tablets (CPIT) (Roche)) at 20 OD units per mL. To the resuspended cells, 5 μg zymolyase 100-T (Nacalai-Tesque) were added per OD unit of yeast. Zymolyase digestion was allowed to proceed for 10 minutes at 30°C with shaking at 200 rpm and monitored as described previously [5]. Spheroplasts were pelleted at 1,500 × g, 4°C for 5 minutes and washed once in spheroplast wash medium (SD-His with the addition of 1.2 M sorbitol and 1 mM EDTA) at 5 OD units per mL.

*Cell lysis for nuclear preparation and density gradient centrifugation*

For the nuclear preparations 120 OD units of cells were used. The nuclear preparation was performed according to [6] with modifications as follows. The spheroplasts were resuspended in 5 mL Ficoll lysis buffer (18% (w/v) Ficoll PM-400 (Sigma), 20 mM dibasic potassium phosphate, pH 6.8, 1.5 mM magnesium chloride, 0.5 mM EDTA, pH 8.0, freshly supplemented with CIPT). Spheroplasts were lysed by 20 strokes in a Dounce homogeniser (Wheaton) on ice. Both lysates were pre-cleared at 3,220 × g for 10 minutes at 4°C. The supernatant was ultracentrifuged at 26,000 RPM, 4°C, in a Type 70 Ti fixed angle rotor (Beckman Coulter), for 35 minutes in an Optima LE-80K ultracentrifuge (Beckman Coulter). The supernatant was discarded and the pellets resuspended in buffer NP (350 mM sucrose, 20 mM Tris-HCl, pH 7.4, 50 mM KCl, 5 mM MgCl_2_, freshly supplemented with CIPT).

*Lysis for cushion and isopycnic density gradient ultracentrifugation*

The remaining spheroplasts (or all spheroplasts, in the experiments performed in the absence of a nuclear preparation) were resuspended in lysis buffer (250 mM sucrose, 10 mM HEPES-NaOH, pH 6.8, 50 mM potassium acetate, 2 mM magnesium acetate, 1 mM EDTA, pH 8.0, CIPT) at 20 OD units per mL. Cell lysis was carried out using a method modified from [7]. Briefly, the spheroplast suspension was transferred to the chamber of a nitrogen cavitation vessel (Model 4639, Parr Instrument Company) which was charged for 3 minutes on ice with oxygen-free nitrogen to a pressure of 500 psi. Pressure was lowered to 300 psi for 3 minutes and the outlet port was opened to discharge the vessel at approximately 3 drops per second.

Lysates were cleared of debris by serial centrifugation at 4°C, for 5 minutes at 1,000 × g, followed by 3,000 × g for 10 minutes. The supernatant was transferred to a round-bottomed polycarbonate ultracentrifuge tube and underlaid with 5 mL 18% (w/v) OptiPrep (Sigma), prepared in lysis buffer. The tube was ultracentrifuged in a SW32Ti rotor (Beckman Coulter) at 28,000 RPM for 2 h at 4°C in a Optima L80-XP ultracentrifuge (Beckman Coulter), with maximum acceleration profile and slow deceleration (profile = 9). The cytosol-enriched supernatant was withdrawn and reserved for downstream processing. The interphase, containing organelle membrane proteins, was withdrawn and the refractive index adjusted to the equivalent of 16% (w/v) OptiPrep with the aid of a handheld refractometer (Bellingham + Stanley). The adjusted membranes were subjected to ultracentrifugation in a VTi65.1 rotor at 65,000 RPM, 4°C for 4 hours with maximum acceleration and slow deceleration (profile = 9). Fractions of approximately 0.5 mL were collected from the density gradient using an Auto-Densiflow peristaltic pump with meniscus-tracking probe (Labconco), from the top of the tube to the bottom.

*Gradient fraction processing*

The protein content of all fractions, including the nuclear fraction, cytosolic supernatant and gradient fractions, was precipitated using trichloroacetic acid (TCA) precipitation at a final concentration of 20% (v/v) TCA at 4°C for 2 hours. Fractions were centrifuged at g_max_ in a micocentrifuge for 10 minutes and the supernatant was discarded before the precipitated protein pellets were washed three times in acetone, with extensive vortexing and sonication. The final wash was discarded and the pellets briefly air-dried before resolubilisation as described previously [8], with the modification that 100 mM HEPES-NaOH, pH 8.5, 0.1% (w/v) SDS was used for resolubilisation. Protein concentrations of all fractions were estimated using a BCA assay (Thermo Scientific) and the distribution of organelles was monitored by western blotting against a panel of organelle markers (Supplementary Figure 3).

Fractions were selected for TMT labelling based on enrichment of organelles of interest, and depletion of contaminating organelles. For experiments involving nuclear preparations, eight fractions were selected from the density gradient for labelling along with the nuclear preparation and cytosolic fraction. For the other experiment, 9 fractions were selected from the density gradient as well as the cytosolic fraction. The protein content in each fraction was normalised to the same amount before reduction by addition of dithiothreitol to 10 mM and heating to 56°C for 1 hour, and subsequent alkylation with iodoacetamide to 25 mM for 2 hours at room temperature in the dark. Fractions were precipitated overnight at -20°C in ten volumes of acetone.

Precipitated fractions were centrifuged at g_max_ in a benchtop microcentrifuge and the supernatant was discarded before fractions were resolubilised in 100 mM HEPES-NaOH, pH 8.5. Proteolytic digestion was carried out at 37°C for 16 hours using Sequencing Grade Modified Trypsin (Promega), at a final ratio of 1:20 (w/w) protease:protein, which was added in two aliquots spaced 1 hour apart.

TMT labelling was carried out as previously described [8]. Pooled samples were subjected to clean-up by solid phase extraction on Sep Pak tC_18_ cartridges (Waters) according to [9] but with the modification that elution was carried out in two steps, using 0.5 mL 75% methanol/0.5% acetic acid followed by 0.5 mL 75% acetonitrile/0.5% acetic acid.

Samples were subjected to pre-fractionation by high pH reversed phase chromatography on a Waters Acquity UPLC system using the mobile phase conditions previously described [1,8]. Peptide-containing chromatographic space was determined and the samples were pooled orthogonally, first with middle, second with second-from-middle and so on until the end of the peptide-containing chromatographic space. Approximately 1 μg of each pooled fraction was analysed by LC-MS/MS using a 120-minute gradient on a Q Exactive mass spectrometer (Thermo Scientific).

*Analysis of samples by liquid chromatography-tandem mass spectrometry (LC-MS/MS)*

LC-MS/MS experiments were performed using a Dionex Ultimate 3000 RSLC nanoUPLC system (Thermo Fisher Scientific Inc., Waltham, MA, USA) coupled in-line to a Q Exactive Orbitrap mass spectrometer (Thermo Fisher Scientific Inc., Waltham, MA, USA). Separation of peptides was performed by reversed phase chromatography at a flow rate of 300 nL/min and a Thermo Scientific reverse-phase nano Easy-spray column (Thermo Scientific PepMap C_18_, 2 μm particle size, 100Å pore size, 75 μm inner diameter (i.d.) x 50 cm length). Peptides were loaded onto a pre-column (Thermo Scientific PepMap 100 C_18_, 5 μm particle size, 100 Å pore size, 300 μm i.d. x 5 mm length) from the Ultimate 3000 autosampler with 0.1% formic acid for 3 minutes at a flow rate of 10 μL/min. The column valve was switched to allow elution of peptides from the pre-column onto the analytical column. Solvent A was water + 0.1% formic acid and solvent B was 80% acetonitrile, 20% water + 0.1% formic acid. The linear gradient employed was 4-40% B in 100 minutes (the total run time including column washing and re-equilibration was 120 minutes).

The LC eluant was sprayed into the mass spectrometer by means of an Easy-spray source (Thermo Fisher Scientific Inc., Waltham, MA, USA). All *m/z* values of eluting ions were measured in an Orbitrap mass analyzer, set at a resolution of 70,000 and scanned between *m/z* 380-1500. Data-dependent scans (Top 20) were employed to automatically isolate and generate fragment ions by higher energy collisional dissociation (HCD, NCE: 32.5%, stepped 10%) in the quadrupole mass analyser and measurement of the resulting fragment ions was performed in the Orbitrap analyser, set at a resolution of 35,000. Peptide ions with charge states of between 2+ and 5+ were selected for fragmentation.

*MS data processing*

Raw data files were processed using Proteome Discoverer version 1.4, interfaced with an in-house Mascot server, and searched against a canonical *S. cerevisiae* database downloaded from UniProt (November 2014; 6,652 sequences). Raw data files corresponding to all fractions from each experiment were merged for searching which yielded a combined results file for the entire experiment. For the Mascot search, a peptide tolerance of ±25 ppm and a fragment match tolerance of ±0.1 Da were allowed. Additional inclusion of the Percolator algorithm [10] node within the software, allowed filtering of peptide-spectral matches based on robust false discovery rate (FDR) determination. Carbamidomethylation of cysteine, and TMT labelling of lysine and peptide N-termini were set as static modifications. Oxidation of methionine, deamidation of asparagine and glutamine, and TMT labelling of serine, threonine and tyrosine were set as variable modifications. For reporter ion quantitation the sum of centroided peaks within a window of ±2 mmu around m/z of each monoisotopic reporter ion was used and MS order for activation was set to MS2. Protein group-level quantitation was set to be equal to the median of all quantified peptide-spectral matches (PSMs) for the protein group in question and protein group quantitation was sum-normalised such that the total intensity across all channels for a protein group was equal to 1. We post-processed our hyperLOPIT data using the R Bioconductor MSnbase [11] package (version 2.2.0). All biological replicates were combined [12] and variance-stabilising normalisation (VSN) [13] was applied to account for differences in technical variation between hyperLOPIT biological replicate experiments whilst maintaining important biological variation.

*Informatics analysis*

*Organelle marker list generation*

Organelle marker proteins used for SVM classification were mined from the *Saccharomyces* Genome Database (SGD) [14] and based on low throughput GO Cellular Compartment (CC) annotation, for which the original literature was consulted. Proteins with a single annotated location were used as markers for protein localisation prediction. To ensure a high quality marker-list high-throughput GO CC annotation was disregarded, and proteins that possessed annotation based solely on high-throughput annotation, were not employed as markers (Supplementary Data 2).

*Protein localisation prediction*

Protein localisation prediction was performed using the R [15] Bioconductor [16] package pRoloc (version 1.21.5) [17] on the combined dataset, where protein groups were quantified in all 4 biological replicate hyperLOPIT experiments. We performed protein classification as in [8,18–20] using a support vector machine (SVM), with weighting of marker classes as the reciprocal of class size to account for class imbalance. Algorithmic performance was estimated using 100 rounds of 5-fold cross-validation, and an additional round of cross-validation on each training partition was conducted to optimise the free parameters of the SVM, sigma and cost, via a grid search. The optimal sigma and cost were chosen to be 0.1 and 8, respectively, based on the best F1 score (the harmonic mean of precision and recall). Protein assignments were manually validated by ranking the SVM scores on an organelle-by-organelle basis and checking for corroboration between SVM classification and low throughput data from the literature contained within the SGD (Supplementary Data 3). To obtain a 5% FDR organelle SVM score cut-offs were set such that the percentage of false assignments was maintained at <5% of the total number of assignments for a specific organelle. Any protein with a score below the cut-off was omitted from assignment to an organelle. If localisation was annotated as “unknown”, was only from high throughput sources, or contained only general annotation such as “membrane” or “intracellular”, this was not counted as a disagreement or agreement with hyperLOPIT.

References

1. Nightingale DJH, Oliver SG, Lilley KS: **Mapping the Saccharomyces cerevisiae spatial proteome with high resolution using hyperLOPIT.** In *Methods in Molecular Biology*. Edited by Oliver SG. Humana Press; 2018:In press.

2. Baker Brachmann C, Davies A, Cost GJ, Caputo E, Li J, Hieter P, Boeke JD: **Designer deletion strains derived fromSaccharomyces cerevisiae S288C: A useful set of strains and plasmids for PCR-mediated gene disruption and other applications**. *Yeast* 1998, **14**:115–132.

3. Huh W-K, Falvo J V, Gerke LC, Carroll AS, Howson RW, Weissman JS, O’Shea EK: **Global analysis of protein localization in budding yeast.** *Nature* 2003, **425**:686–91.

4. Breker M, Gymrek M, Schuldiner M: **A novel single-cell screening platform reveals proteome plasticity during yeast stress responses**. *J Cell Biol* 2013, **200**:839–850.

5. Rieder SE, Emr SD: **Isolation of Subcellular Fractions from the Yeast Saccharomyces cerevisiae**. *Curr Protoc Cell Biol* 2001, **8**:1–68.

6. Kizer KO, Xiao T, Strahl BD: **Accelerated nuclei preparation and methods for analysis of histone modifications in yeast**. *Methods* 2006, **40**:296–302.

7. Wang Y, Lilley KS, Oliver SG: **A protocol for the subcellular fractionation of Saccharomyces cerevisiae using nitrogen cavitation and density gradient centrifugation.** *Yeast* 2014, **31**:127–35.

8. Christoforou A, Mulvey CM, Breckels LM, Geladaki A, Hurrell T, Hayward PC, Naake T, Gatto L, Viner R, Arias AM, et al.: **A draft map of the mouse pluripotent stem cell spatial proteome**. *Nat Commun* 2016, **7**:9992.

9. Villén J, Gygi SP: **The SCX/IMAC enrichment approach for global phosphorylation analysis by mass spectrometry.** *Nat Protoc* 2008, **3**:1630–8.

10. Brosch M, Yu L, Hubbard T, Choudhary J: **Accurate and sensitive peptide identification with mascot percolator**. *J Proteome Res* 2009, **8**:3176–3181.

11. Gatto L, Lilley KS: **Msnbase-an R/Bioconductor package for isobaric tagged mass spectrometry data visualization, processing and quantitation**. *Bioinformatics* 2012, **28**:288–289.

12. Trotter MWB, Sadowski PG, Dunkley TPJ, Groen AJ, Lilley KS: **Improved sub-cellular resolution via simultaneous analysis of organelle proteomics data across varied experimental conditions.** *Proteomics* 2010, **10**:4213–9.

13. Huber W, von Heydebreck A, Sültmann H, Poustka A, Vingron M: **Variance stabilization applied to microarray data calibration and to the quantification of differential expression.** *Bioinformatics* 2002, **18 Suppl 1**:S96-104.

14. Cherry JM, Hong EL, Amundsen C, Balakrishnan R, Binkley G, Chan ET, Christie KR, Costanzo MC, Dwight SS, Engel SR, et al.: **Saccharomyces Genome Database: the genomics resource of budding yeast.** *Nucleic Acids Res* 2012, **40**:D700-5.

15. R Core Team: **R: A Language and Environment for Statistical Computing.** 2017, URL http://www.R-project.org/.

16. Gentleman RC, Carey VJ, Bates DM, Bolstad B, Dettling M, Dudoit S, Ellis B, Gautier L, Ge Y, Gentry J, et al.: **Bioconductor: open software development for computational biology and bioinformatics.** *Genome Biol* 2004, **5**:R80.

17. Gatto L, Breckels LM, Wieczorek S, Burger T, Lilley KS: **Mass-spectrometry-based spatial proteomics data analysis using pRoloc and pRolocdata**. *Bioinformatics* 2014, **30**:1322–1324.

18. Breckels LM, Mulvey CM, Lilley KS, Gatto L: **A Bioconductor workflow for processing and analysing spatial proteomics data**. *F1000Research* 2016, **5**:2926.

19. Mulvey CM, Breckels LM, Geladaki A, Britovšek NK, Nightingale DJH, Christoforou A, Elzek M, Deery MJ, Gatto L, Lilley KS: **Using hyperLOPIT to perform high-resolution mapping of the spatial proteome**. *Nat Protoc* 2017, **12**:1110–1135.

20. Thul PJ, Åkesson L, Wiking M, Mahdessian D, Geladaki A, Ait Blal H, Alm T, Asplund A, Björk L, Breckels LM, et al.: **A subcellular map of the human proteome.** *Science* 2017, **356**:eaal3321.
